# Facial thermal variations: A new marker of emotional arousal

**DOI:** 10.1371/journal.pone.0183592

**Published:** 2017-09-18

**Authors:** Vladimir Kosonogov, Lucas De Zorzi, Jacques Honoré, Eduardo S. Martínez-Velázquez, Jean-Louis Nandrino, José M. Martinez-Selva, Henrique Sequeira

**Affiliations:** 1 School of Psychology, University of Murcia, Murcia, Spain; 2 Academy of Psychology and Educational Sciences, Southern Federal University, Rostov-on-Don, Russia; 3 SCALab, UMR 9193, CNRS & University of Lille, Lille, France; 4 Institute of Neuroscience, University of Guadalajara, Guadalajara, Mexico; 5 Facultad de Psicología, Meritorious Autonomous University of Puebla, Puebla, Mexico; 6 Murcia Institute for Biomedical Research (IMIB-Arrixaca), Murcia, Spain; Universita degli Studi di Udine, ITALY

## Abstract

Functional infrared thermal imaging (fITI) is considered a promising method to measure emotional autonomic responses through facial cutaneous thermal variations. However, the facial thermal response to emotions still needs to be investigated within the framework of the dimensional approach to emotions. The main aim of this study was to assess how the facial thermal variations index the emotional arousal and valence dimensions of visual stimuli. Twenty-four participants were presented with three groups of standardized emotional pictures (unpleasant, neutral and pleasant) from the International Affective Picture System. Facial temperature was recorded at the nose tip, an important region of interest for facial thermal variations, and compared to electrodermal responses, a robust index of emotional arousal. Both types of responses were also compared to subjective ratings of pictures. An emotional arousal effect was found on the amplitude and latency of thermal responses and on the amplitude and frequency of electrodermal responses. The participants showed greater thermal and dermal responses to emotional than to neutral pictures with no difference between pleasant and unpleasant ones. Thermal responses correlated and the dermal ones tended to correlate with subjective ratings. Finally, in the emotional conditions compared to the neutral one, the frequency of simultaneous thermal and dermal responses increased while both thermal or dermal isolated responses decreased. Overall, this study brings convergent arguments to consider fITI as a promising method reflecting the arousal dimension of emotional stimulation and, consequently, as a credible alternative to the classical recording of electrodermal activity. The present research provides an original way to unveil autonomic implication in emotional processes and opens new perspectives to measure them in touchless conditions.

## Introduction

Emotional states involve brain processes and body reactions. Within the repertoire of physiological and behavioral responses, autonomic ones constitute important markers of these states [1, 2, 3]. Indeed, several autonomic variables have proved to index subjective dimensions of emotion, like valence (pleasant vs. unpleasant) and arousal (low vs. high) [4]. Moreover, these variables can hardly be voluntarily controlled, contrary to behavioral responses involving skeletal muscles (postures, gestures, mimicries) and can therefore bring significant information beyond usual verbal and nonverbal behaviors [5].

The facial expression of emotions, including autonomic responses such as pallor, blush, or pupil size, takes part in interpersonal communication by informing congeners about threatening or attractive events the individual is faced with. One challenge for affective sciences is to measure facial correlates of spontaneous emotional arousal, i.e. to obtain recordings of facial autonomic cues with distant touchless apparatus. In recent years, functional infrared thermal imaging (fITI) of the face has emerged as a promising non-invasive tool for the study of the autonomic response to emotions [6, 7]. Facial thermal response proved to be modulated by various aspects of social interactions likely to affect emotional state, such as interpersonal distance or partner’s gender [8, 9, 10, 11, 12]. It also appeared to vary with emotional content of stimuli occurring in non-social contexts ([13], for a review).

fITI remotely captures cutaneous surface thermal radiation which depends on cutaneous blood perfusion controlled by the autonomic nervous system (ANS) innervating the vessels that irrigate the skin (see [13]). Though the parasympathetic system has an influence through the endothelial cells, in glabrous skin (palmar and plantar surfaces, tip of the nose), the vasomotion appeared regulated principally by sympathetic noradrenergic fibers, whose activation leads to vasoconstriction and, therefore, to a decrease in local temperature [14, 15]. In particularly, the tip of the nose seems to present consistent thermal variations in response to emotional activation [13]. This could be explained by the absence of underlying muscles, which avoids thermal contamination due to contraction, and by the presence of abundant arteriovenous anastomoses in this glabrous area involved in the regulation of body temperature [16, 17]. Indeed, decreases in nose temperature were found with both pleasant (joy) and unpleasant (disgust, fear, anger, sadness) information [18, 19, 20]. For several authors the nose thermal changes are nonspecific regarding negative or positive situations [21, 22, 23] which in terms of the dimensional theory of Lang and co-workers [4] suggests that these decreases are indicative of emotional arousal. In line with this view, decrease in nose temperature has also been correlated with central arousal measures, e.g., alpha rhythm attenuation [24].

Emotional activation seems to be the relevant dimension able to modulate ANS output: the higher an emotion’s intensity, the greater the ANS activation [25]. In this respect, it has repeatedly been shown that autonomic activation reflects emotional arousal through a large spectrum of autonomic measures, e.g. skin conductance [26] and gastric myoelectrical activity [27]. Considering the sympathetic influence on thermal variations, it could be expected that these variations would depend on emotional arousal as well. However, in order to assess the potentiality of fITI as a marker of emotional arousal, an alternative to the presentation of discrete emotions used in the majority of the previous studies appears preferable. Indeed, the presentation of standardized pictures with known values of valence and arousal, proved to be an efficient approach with other autonomic indices such as the electrodermal activity (EDA) [28]. The EDA, which is related to the activity of eccrine sweat glands exclusively innervated by the sympathetic branch of the ANS, is known to be a robust index of central arousal linked to attention, novelty and especially emotion. In fact, following an emotional stimulation, electrodermal variation amplitude, usually recorded as skin conductance responses (SCRs) in palms, increases with the subjective arousing value of the emotional stimulus, regardless of affective valence [4, 26]. SCRs are widely employed measures when studying the arousal dimension of emotions and constitute reliable autonomic markers of emotional arousal and its somato-visceral impact [4, 28].

As a consequence, it appears advisable to include the simultaneous measure of skin conductance and face thermal imprints in order to better characterize fITI as a measure of emotion. A few studies brought some information on this topic [29, 30, 22]. The perinasal temperature response proved to be nonspecific regarding negative or positive situations and electrodermal responses. In the work by Di Giacinto et al. [29] an increase in tonic skin conductance activity and a decrease in nose temperature occurred in patients with post-traumatic stress disorder in comparison with healthy controls. These findings are in line with those of Coli et al. [31] who showed that facial thermal responses and SCRs have similar ability to reveal emotional variations although their latencies may differ. Finally, these data as well as the fact that thermal and electrodermal variations could be evoked by emotional situations, in animals [30] and in humans [7], favour the hypothesis that both thermal and electrodermal variations can index emotional arousal. Nevertheless, in spite of the above studies, we lack data combining subjective judgments, fITI and electrodermal measures and using standardized stimuli based on the dimensional theory of emotions.

The aim of the present study was three-fold: 1) to test the influence of emotional arousal on facial temperature; 2) to compare facial thermal responses with subjective evaluations of arousal and with electrodermal responses, a robust index of emotional arousal; 3) to investigate the existence of a thermo-dermal pattern as a specific expression of sympathetic output to the skin. To this end, standardized emotional pictures differing only in arousal level (low or high activation) and in valence (unpleasant, neutral, pleasant) were presented to healthy participants and behavioral (subjective emotion ratings) and autonomic (skin thermal and dermal responses) responses were recorded. The nose region was used as the region of interest for recording thermal responses. Moreover, considering that emotional arousal rates are positively correlated with sympathetic activation [4, 32, 33], we expected both decreases in nasal skin temperature (due to vasoconstriction) and increases in skin conductance related to the arousal evaluation of the emotional pictures presented to the participants. Furthermore, analysis of thermal and electrodermal signals was expected to show a convergent pattern of reactivity, with a common dependence from sympathetic output.

## Methods

### Participants

The study was approved by the Ethics Committee of the University of Lille 3. The persons suffering from neurologic or psychiatric diseases were excluded like those taking drugs interfering with the adrenergic system. Twenty four student volunteers (19 females) from the University of Lille participated in the study. They were 22.4 ± 1.8 years old (mean ± SD). The sample size is in line with those of recent studies that used nose thermography [8, 19, 21, 29, 34].

**Fig 1:**
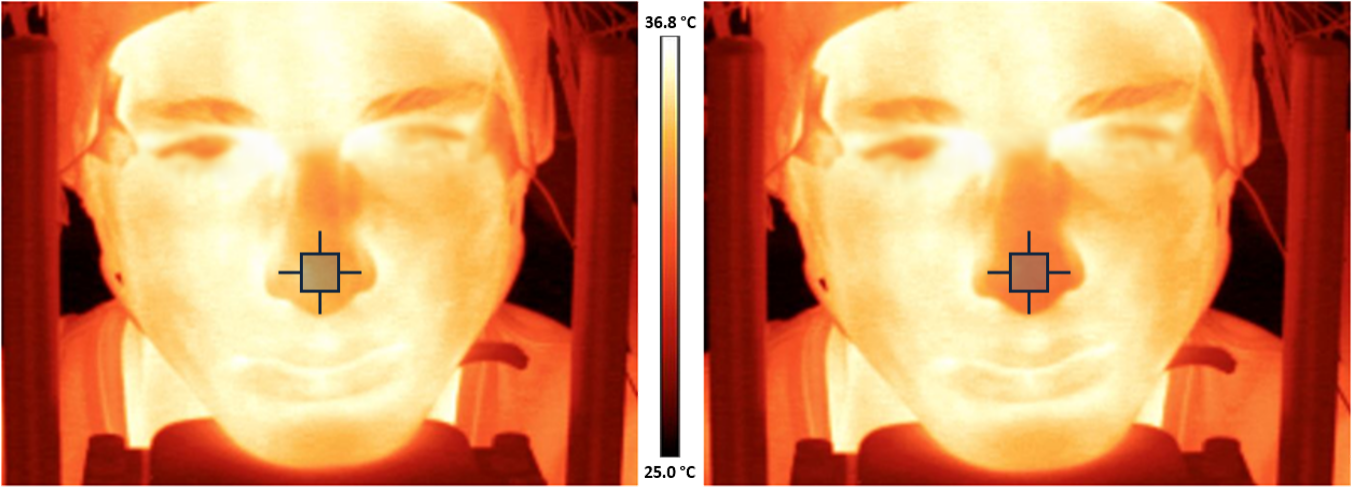
Examples of thermal images during rest (left) and during unpleasant visual stimulation (right). Note the temperature decrease in the nose region to the stimulation. Temperature scale in °C (from 25 to 36.8).

All the participants gave written informed consent in accordance with the Declaration of Helsinki and were asked to avoid coffee, tobacco or alcohol consumption the day of the experiment. Their vision was normal or corrected-to-normal. In the later case they were asked to wear contact lenses. The individual appearing in the Fig 1 has given written informed consent (as outlined in PLOS consent form) to publish thermal images.

### Stimuli

We selected 60 pictures (20 pleasant, 20 unpleasant, and 20 neutral) from the International Affective Picture System (IAPS; [35]). The IAPS values for the pictures were the following: valence = 2.27 ± 1.40, and arousal = 5.77 ± 2.21 for unpleasant pictures; valence = 4.97 ± 1.15, and arousal = 2.91 ± 1.91 for neutral pictures; valence = 7.33 ± 1.53, and arousal = 5.43 ± 2.23 for pleasant pictures. Comparisons with the Student t-test confirmed that the pleasant pictures had greater valence scores than neutral ones and neutral pictures had greater scores than unpleasant ones (all ps < .001). There were no significant differences in arousal between pleasant and unpleasant pictures (p = .16) and both had greater arousal than neutral ones (all ps < .001).

### Recordings

An infrared thermal imaging camera FLIR SC5000 (FLIR systems, USA) and a Research IR 4.0 software were used to record temperature. The spatial resolution was 320 × 256 ppi and the temperature resolution, 0.01°C. The emissivity was set at 0.98 [36]. The recording frequency was 30 Hz and we reduced it off-line till 1 Hz to extract values for processing. We analyzed the mean temperature of a square (3 × 3 pixels) on the tip of the nose (Fig 1).

Skin conductance responses were obtained through bipolar Ag/AgCl standard surface electrodes filled with an isotonic electrolyte paste and placed on the index and middle fingers of the non-dominant hand of the participant. SCRs were recorded using a Biopac MP150 system at a sampling rate of 500 Hz, amplified with a gain of 5 μΩ / V and low-pass filtered at 10Hz. The raw signal was calibrated to detect activity in the 0–100 μS range. The threshold amplitude change for any wave to be considered a SCR was .05 μS. At the end of each set, the participant was asked to look again at all the pictures and to rate the valence and arousal values using a nine-point SAM scale (Self-Assessment Manikin [37]), ranging from 1, very unpleasant, to 9, very pleasant, and from 1, very calm, to 9, very arousing. Ratings were recorded with an OpenSesame program [38].

### Procedure

**Fig 2:**
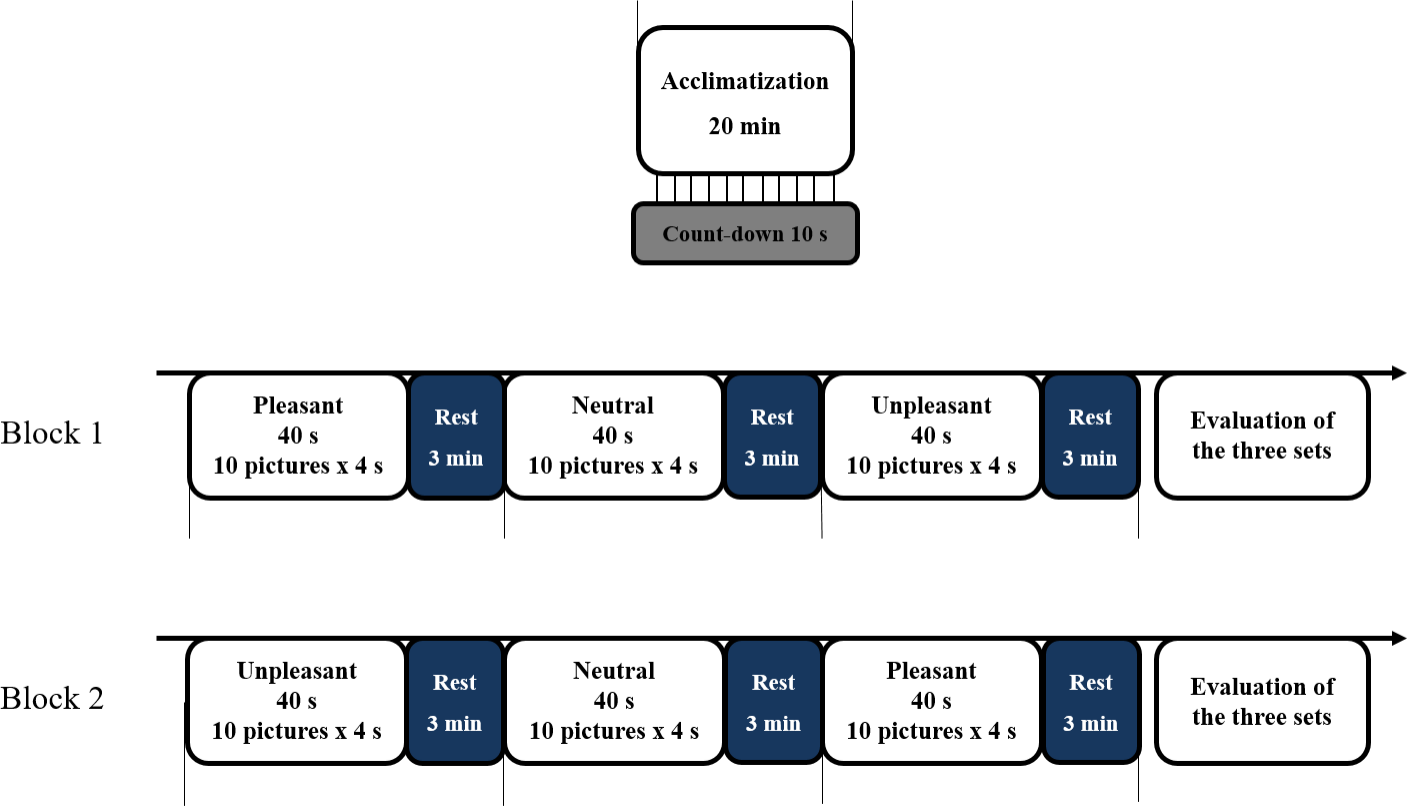
Design of the study. Note that half of the participants started with the pleasant condition (block 1) and the other half with the unpleasant condition (block 2). The dashes represent a 10 s count-down before each set. One trial consisted in the consecutive presentation of ten pictures, followed by a 3-min resting time.

The experiments were carried out in the morning, between 9 and 12. Room temperature in the laboratory throughout the study was held constant, at 24°C. A 20 min acclimatization period was planned before the experiment. Participants were seated in an armchair with chin resting on a support and forehead leaning on a horizontal stick of the support. They were asked to avoid head movements and to passively observe the pictures which were presented on a 50 cm monitor located 1.2 m from the head support. The pictures were divided into six sets, each comprising 10 pictures of the same valence, distributed into block 1 and block 2 corresponding to two different presentation orders: pleasant–neutral–unpleasant and unpleasant–neutral–pleasant (Fig 2). One trial consisted in the consecutive presentation of the ten pictures of a set for 40 s (each picture lasted 4 s), followed by an interset resting interval of 3 min allowing the thermal response to build up. After each block, the participants were asked to rate the affective valence and arousal of the entire sets. The order of the blocks was counterbalanced across participants.

### Data analysis

**Fig 3:**
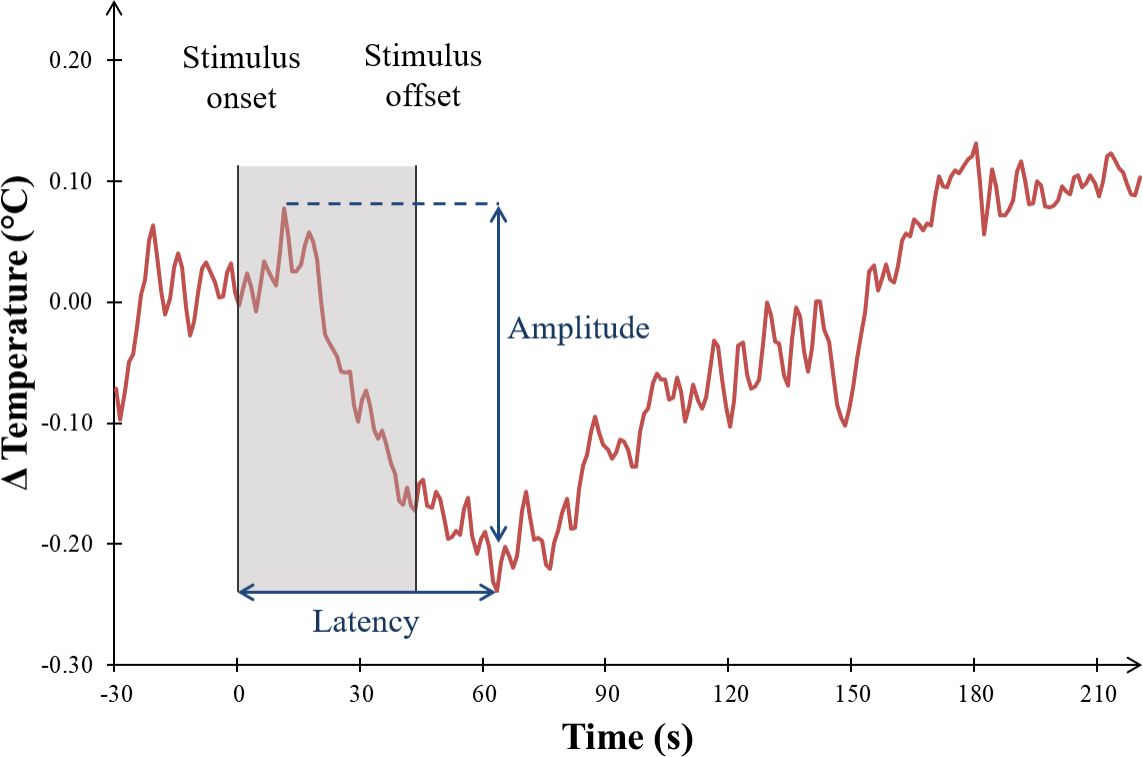
A typical thermal response in an unpleasant trial. Thermal amplitude was defined as the difference between the first maximum during the stimulation and the following minimum, and the latency as the difference between stimulus onset and the same minimum.

Thermal data were smoothed using the weighted arithmetic mean algorithm with 5 weights (1, 2, 3, 2, 1). To minimize the impact of long-time spontaneous fluctuations, linear variations between the beginnings of consecutive sets were subtracted from the signal. Then all the trials of each participant were visually examined, by two authors (VK and LDZ) independently. In some trials, it was not possible to detect any response and the amplitude was set to zero. When a response was detected, the first maximum during the stimulation and the minimum during the stimulation or the subsequent period of rest were selected and the difference between these two values was calculated to obtain the amplitude of change (see Fig 3). The smallest detected response had an amplitude of—0.035°C. The peak latency was the time between the stimulus onset and the maximum temperature change during the stimulation or the rest. In the trials with no detectable response, the latency could not be determined.

SCRs were computed as the highest value of the wave appearing 1 s after the onset of the first picture of each set and having an amplitude superior or equal to 0.05 μS [39]. SCRs occurring during the stimulation period were accounted and the amplitude of these responses were summed and taken as an index of electrodermal reactivity to affective pictures. All SCRs occurring during the stimulation period were accounted for and two parameters expressing total electrodermal activity were calculated, i.e. frequency (number of SCRs per minute) and mean of their amplitudes.

### Statistical analysis

In line with the dimensional framework, the effect of emotional conditions, unpleasant (U), neutral (N) and pleasant (P), was broken into two components, a valence and an arousal effects (see [40]. The valence effect was calculated as the difference [U—P], i.e., by applying a linear contrast (LC) to the factor emotion. The activation effect was calculated as the difference [(U + P) / 2)—N], which is obtained by applying a quadratic contrast (QC). The two contrasts are orthogonal, which means that the valence and arousal effects are independent and can be analyzed separately. Besides, they explain the whole variability associated to the factor emotion. When the linear contrast only is significant, the emotion has a pure valence effect, when the quadratic contrast only is significant, it has a pure activation effect. When both are significant one may suspect a negativity or positivity bias, when none is significant, emotion has no effect on the dependent measure.

The distributions of thermal and thermal amplitudes included a proportion of zeroes which kept them from normality. This had two consequences on the analysis strategy we adopted. Firstly, non-parametric tools were preferred, and their use was extended to the subjective ratings in order to conclude from the same kind of tools for all the dependent measures. Thus, the valence (U vs P) and arousal ((U + P) / 2 vs N) contrasts were assessed with the Wilcoxon T-test. Secondly, we took advantage of this recurrent difficulty (for example, habituation of EDA is well documented in the literature; [41]) by analyzing in detail the frequency of trials with and without thermal and dermal responses, in order to identify physiological response patterns using Yates X^2^ test. We also performed correlation analyses (Spearman's rho) between subjective evaluations and physiological parameters using rank (rk) differences corresponding to the above contrasts, i.e. rk(U)—rk(P) for valence and rk(UP)—rk(N) for arousal.

All the analyses were carried out with Statistica software and all tests used a .05 significance level.

## Results

### Subjective ratings

**Fig 4:**
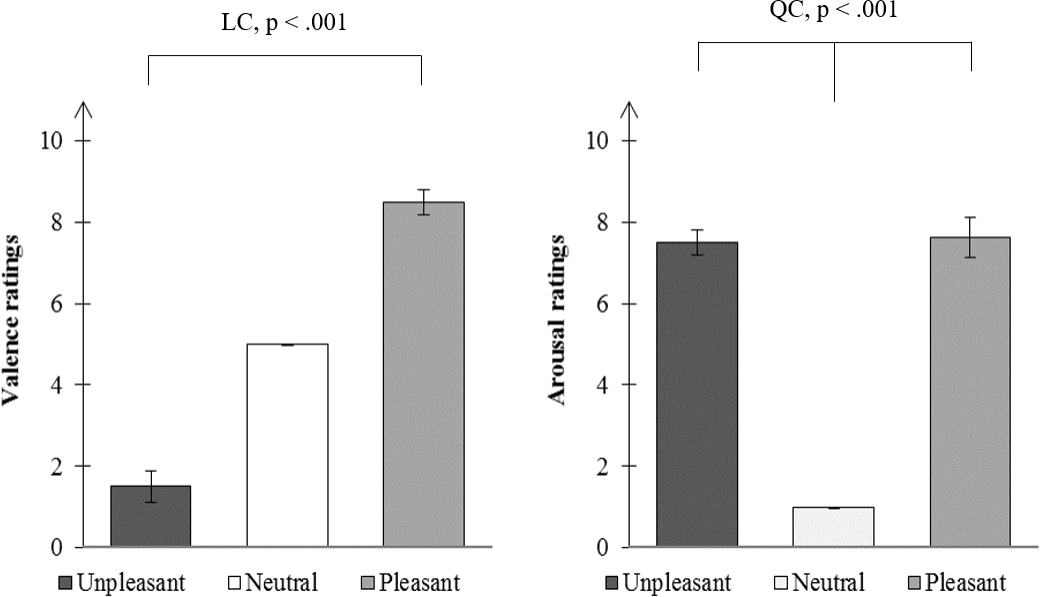
Median subjective ratings for the pictures’ valence (left) and arousal (right). The error bars represent the Semi Inter-Quartile Range (SIQR). LC, linear contrast; QC, quadratic contrast.

As expected, the analysis of valence ratings showed a valence effect (T = 0; N = 24; p < .001) but no arousal effect (T = 65.5; N = 24; p = .636). Reciprocally, the analysis of arousal ratings showed an arousal effect (T = 0; N = 24; p <. 001) but no valence effect (T = 96; N = 24; p = .322). The median ratings are given in Fig 4.

### Thermal responses

**Fig 5:**
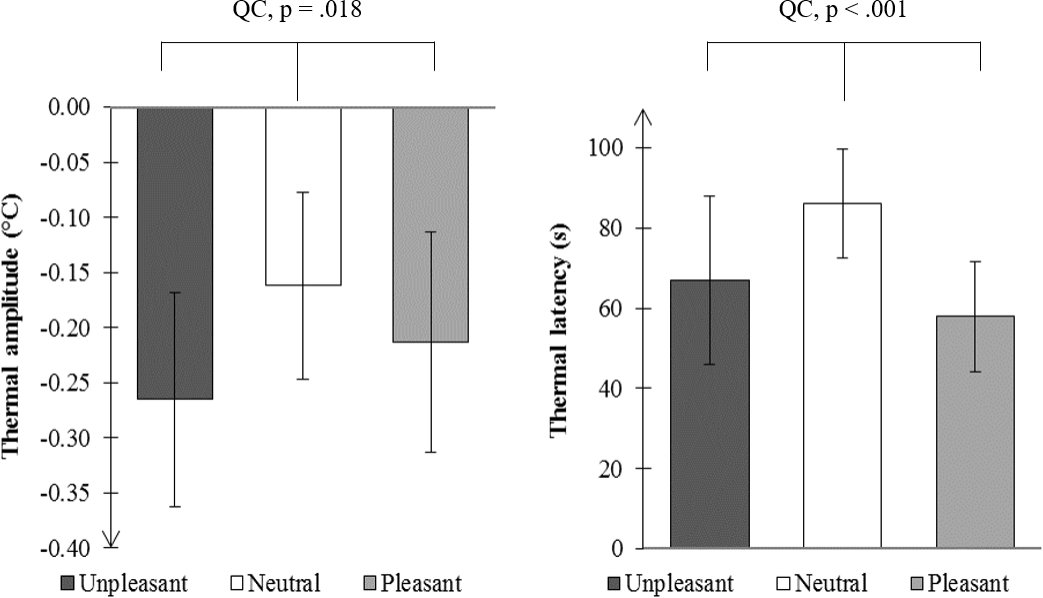
Medians and the SIQR of thermal amplitude (left) and latency (right) for unpleasant, neutral and pleasant stimulations. QC, quadratic contrast.

We found an arousal effect of emotional pictures on the amplitude of the temperature decrease (T = 67; N = 24; p = .018, Fig 4), but no valence effect (T = 107.5; N = 24; p = .225). The outcome was the same for latency analysis (Fig 5): emotional pictures shortened the latency of the temperature peak (T = 2; N = 16; p < .001) and there was no difference between U and P conditions (T = 64; N = 16; p < .836). We also observed a negative correlation between QCs (arousal effects) calculated for subjective arousal ratings and for amplitudes of thermal responses (rho = -.60; df = 22; p = .002).

### Electrodermal responses

**Fig 6:**
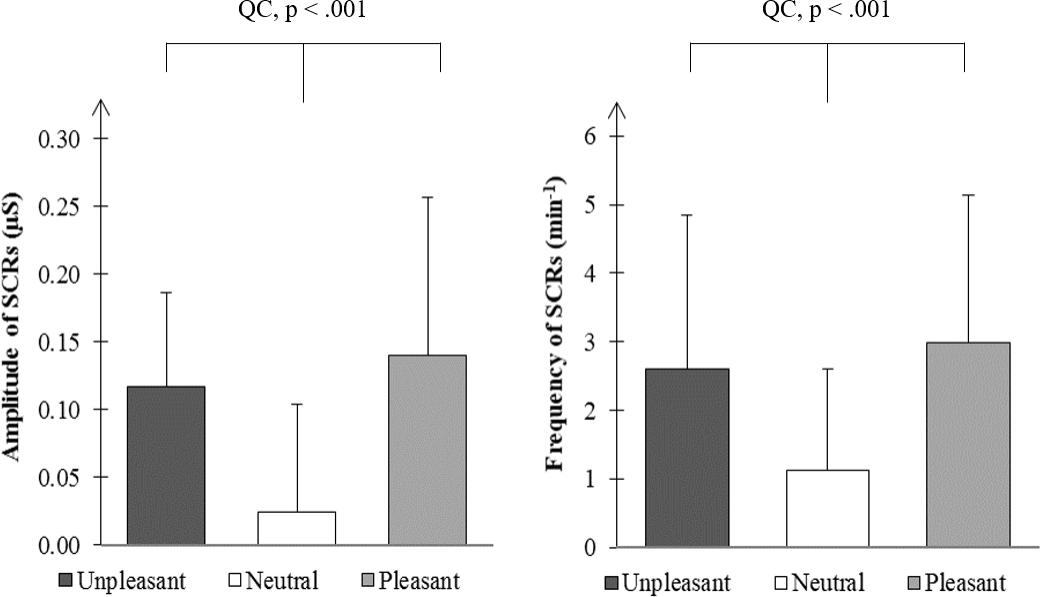
Medians and SIQR of skin conductance responses (SCRs) amplitude (left) and frequency (right) for unpleasant, neutral and pleasant stimulations. QC, quadratic contrast.

Unpleasant and pleasant pictures were associated with higher SCRs frequency than neutral pictures (Fig 6). We found an arousal effect of emotional pictures on the mean of SCRs amplitude (T = 17; N = 24; p < .001), but no valence effect (T = 120; N = 24; p = .584). An arousal effect of emotional pictures was also found on SCRs frequency (T = 0; N = 24; p < .001), but not a valence effect (T = 129; N = 24; p = .789). QCs calculated on SCR amplitudes tended to correlate positively with that calculated on subjective ratings (rho = -.35; df = 22; p = .094). QCs for SCR and thermal amplitudes did not correlate (rho = -.05; df = 22; p = .827). However, SCR and thermal amplitudes did correlate when emotional conditions (U and P) where considered without the neutral one (rho = -.43; df = 22; p = .034).

### Patterns of physiological responses

There were trials without detectable thermal (29%) or dermal (41%) responses, especially in the neutral condition (46% and 58%, respectively). The trials without responses were less numerous in the first block than in the second one. The difference was small for the thermal signal (28% vs 31%), more marked for the dermal signal (33% vs 49%).

**Fig 7:**
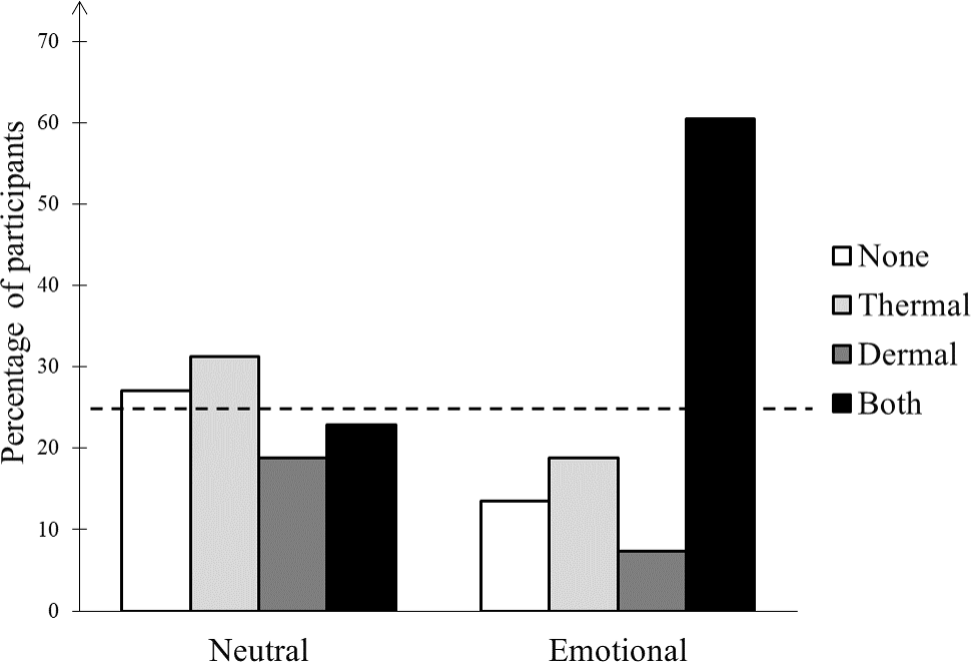
Percentage of participants expressing the physiological patterns of thermal and electrodermal responses in neutral (N) and emotional (mean of P and U) conditions for both blocks. The dashed line represents the equiprobability hypothesis.

The percentages of participants presenting with each of the four possible patterns of response (‘none’, no thermal nor dermal responses; ‘thermal’, thermal response only; ‘dermal’, electrodermal response; ‘both’, thermal and dermal responses; Table 1 and Fig 7) did not differ in the neutral condition (X^2^ Yates = 0.33; df = 3; p = 0.954). However, in the emotional conditions (P and U), the distribution of these percentages differed from the equiprobability observed in the neutral condition (X^2^ Yates = 14.02; df = 3; p = 0.003), without difference between the distributions obtained in P and U conditions (X^2^ Yates = 0.22; df = 3; p = 0.974). A further comparison of 'only' (thermal or dermal) vs 'both' (thermal and dermal) patterns showed that the frequency of the 'only' patterns decreased in the emotional conditions while that of the 'both' pattern increased (X^2^ Yates = 5.60; df = 1; p = 0.018). Finally, the observed distributions were compatible with the hypothesis that dermal and thermal responses did not depend on each other, for both the neutral (X^2^ Yates < .01; df = 1; p = .962) and the emotional conditions (X^2^ Yates = 1.81; df = 1; p = 0.179).

**Table 1:** Percentages of participants showing each pattern of response in the three emotional conditions (means of the two blocks).

	None	Thermal	Dermal	Both	X^2^ Yates	df	p
**Pleasant**	16.7	18.8	6.3	58.3	12.58	3	**0.006**
**Neutral**	27.1	31.3	18.8	22.9	0.33	3	**0.954**
**Unpleasant**	10.4	18.8	8.3	62.5	15.75	3	**0.001**

## Discussion

The aim of this study was firstly, to evaluate the capacity of the facial thermal responses to reveal emotional dimensions of visual stimuli; secondly, to compare the thermal reactivity to subjective and electrodermal responses, commonly used to reveal the same dimensions; finally, to characterize thermal and dermal responses as the sympathetic output associated with central emotional arousal. Based on the sympathetic nature of thermal and electrodermal responses, we predicted that the presentation of emotional pictures would result in an activation effect on these indices: the more arousing the pictures, the greater the decrease in nasal skin temperature and the increase in electrodermal activity. A correlation between the subjective evaluation and these physiological responses was also expected. In support of our predictions, we confirmed the emotional arousal impact of pictures on electrodermal responses, we found a specific influence of such arousal on the facial thermal reactivity, and showed a specific thermo-dermal pattern of reactivity to emotional pictures when compared to neutral ones.

### Emotional value of stimuli

Before measuring the emotional impact of pictures on facial thermal reactivity, the emotional value of these pictures was carefully evaluated on the basis of the dimensional theory, which considers that this value has two dimensions i.e., the valence and the arousal dimensions [4]. In the present study, emotional value was obtained from individual subjective ratings and related to electrodermal measures. The subjective assessment of the three emotional categories (unpleasant, neutral and pleasant) differed in valence while, in arousal, pleasant and unpleasant pictures did not differ from each other but differed from neutral ones. Thus, participants clearly distinguished pleasant from unpleasant pictures and both kinds of pictures from neutral ones, thus validating our picture selection in terms of emotional valence and arousal. On the other hand, our results are consistent with classical and robust results showing no significant electrodermal difference as a function of valence and distinct electrodermal variations as a function of emotional arousal [28, 32, 33]. Indeed, both amplitude and frequency values of SCRs were greater for emotional than for neutral stimuli, but did not differ between unpleasant and pleasant pictures. The emotional arousal effect on SCRs is in agreement with an extensive literature showing that electrodermal activity is a indicator of choice of reticulo-cortical activation (see reviews of [42], and [43]) associated to attention, action preparation and emotional central processes [28, 44]. Furthermore, electrodermal signals’ reliability in revealing central arousal has been extended to subliminal reticular or emotional stimulation. In fact, reticular stimulation was shown to elicit SCRs at intensities lower than the threshold necessary to evoke an EEG arousal in animals [45] and it was also demonstrated that human participants can produce large SCRs to emotional stimuli they do not overtly discriminate [46]. As far as emotional valence is concerned, to the best of our knowledge, electrodermal measures seem unable to reveal significant differences between pleasant and unpleasant stimulations, contrary to other autonomic indices such as heart rate variations [41]. Finally, the fact that the arousal effects of pictures on SCRs amplitude tended to correlate with their subjective ratings is in accordance with the literature [32, 33] and further grounds SCRs as a reliable index of central activation subtending emotion. Overall, in this study, subjective ratings and electrodermal measures contribute to validate the emotional value of stimuli in terms of valence and arousal dimensions.

### Facial thermal imaging: A marker of emotional arousal

The mean temperature decreases obtained in this study are in the range of those obtained in other studies [18, 47]. Indeed, the highest temperature decrease was of about -0.25°C, and the latency after stimulus onset of the highest decrease of about 60 s for the pictures with high levels in arousal. These values are similar to those obtained by Nakayama et al. [48] and by Kuraoka and Nakamura [30] which can be considered as an indicator of the reliability of our data. Besides, an activation effect of emotional pictures was found on amplitude and latency of nasal thermal responses without valence effect: the more arousing the pictures, the faster and larger the thermal responses. This result parallels the one obtained by Ioannou et al. [34] in their study of guilt in children where they found that the higher the distress signs, the higher the decrease in nose temperature. However, while Ioannou and colleagues studied thermal variations induced by guilt in children, we studied thermal variations induced by emotional (unpleasant and pleasant) and neutral pictures. In accordance with the dimensional theory of emotions, the physiological spillover effect from the emotion to the neutral condition (arousal effect) and that from unpleasant to pleasant condition (valence effect) were analyzed. We found an arousal effect without valence effect, indicating that responses to emotional pictures were different from responses to neutral pictures; in addition, there was no difference between responses to unpleasant pictures and responses to pleasant ones. This is in accordance with the findings of Pavlidis et al. [22] and indicates that facial thermal imaging is a reliable non-invasive technique for the assessment of emotional arousal, at least in the framework of the dimensional theory [4]. Contrasting results regarding the arousal dimension and nose thermal changes have been reported by Salazar-Lopez et al [23], who found that high arousal images from the IAPS elicited temperature increases on the tip of the nose, while for low arousal images there were still increases for pleasant images and decreases for unpleasant ones. For the reason that in the current study the low level of arousal corresponds to neutral images, the only possibility to compare both studies is to take into account high arousal images. Yet, for these images we observed temperature decreases, contrary to temperature increases reported by Salazar-López et al. [23], Differences in the procedure and lack of an independent measure of arousal in the study of Salazar-López et al. [23] prevent a direct comparison of results. In additon, when studies of the literature are considered, it can be concluded that our results are more in line with temperature decreases observed in most protocols including different degrees of emotional activation (e.g. [13]).

Considering the short latencies and high amplitudes to arousing pictures, we suggest that the faster and greater temperature decrease to these pictures (pleasant and unpleasant) reveals autonomic adjustments typically associated with emotional situations. The induced sympathetical vasocontrictive reaction has many adaptive functions in the context of the fight/flight response, in particular that of redistributing blood flow. This generally corresponds to an increase in muscle irrigation favouring a rapid response and a decrease in skin surface irrigation that could reduce the effects of injuries in the course of an aggressive encounter. Another consequence of this reaction is that the face becomes paler which may signal an emotional state to congeners [49] indicating the presence of something dangerous, relevant or useful in the environment.

Finally, there were as many trials without thermal responses in the second block than in the first one, suggesting that thermal activity does not undergo rapid habituation, as compared to dermal activity, allowing the thermal technique to be employed when different emotional stimuli must be presented in a short amount of time. If confirmed by further investigations, this could constitute a methodological advantage, in addition to its non-invasive and contact-free nature. This technical advantage could be particularly useful when emotional and other arousing conditions need to be studied from a certain distance or in clinical settings when the patients have difficulties with bodily contact as it happens with severe disturbances like autism.

### Thermal versus dermal responses: A double autonomic signature for emotional arousal

While nasal temperature decreases in response to emotional stimulation, electrodermal activity increases, both responses reflecting the arousal dimension of emotional stimulation. This is coherent with the fact that thermal reactions were negatively correlated with the subjective evaluation of arousal whereas electrodermal responses tended to be positively associated with the same subjective ratings. The fact that the correlation between the arousal measures derived from the SCRs and the subjective evaluations of stimulation was just a trend could be explained by the temporal characteristics of our stimulation, i.e. series of ten 4-s pictures each without any interstimulus interval, which may not be optimal for the collection of electrodermal activity [44]. These findings would also indicate that face temperature changes are more sensitive to subjective emotional arousal than skin conductance ones.

Neurally, changes in both responses reveal the sympathetic activation mediating the central arousal: the thermal decrease probably reflects subcutaneous vasoconstriction [13] and the electrodermal increase is elicited by the activation of sweat eccrine glands (see [28]). It is to note that, though there was no correlation between thermal and dermal amplitudes when the arousal effect was considered, a negative correlation did emerge when emotional conditions (unpleasant and pleasant) where considered without neutral one. Taken together, these correlations could be explained by the fact that the patterns of response appeared random in the neutral condition: some participants presented thermal but no dermal responses, or dermal but no thermal responses. Also, skin conductance and thermal changes in the nose depend on sympathetic activity but their physiological bases are different: while the physiological regulation of the electrodermal activity depends on cholinergic sympathetic fibers, the physiological regulation of the vasomotion of the tip of the nose seems to depend on noradrenergic sympathetic fibers. Finally, the data suggests that a correlated activation of these two systems is detectable only when a threshold level of arousal is exceeded.

Our study showed that the percentage of thermal or dermal 'only' responses was lower in emotional conditions while the frequency of 'both' responses was strongly increased. It also suggested that thermal and dermal subsystems functioned independently in response to neutral scenes and at the mild emotional arousal levels that correspond to the IAPS scenes. In this frame, it can be also suggested that, for high emotional arousal levels, a descending control will generate enough activations, mainly by reticulo-spinal pathways [42], to conjugate discharges of both autonomic channels. This is consistent with the results of Bär et al. [50] showing that sympathetic output from central autonomic network is more uniform during stress and emotion conditions. Furthermore, this response pattern matches the view that emotional arousal can be differentiated per se, based on autonomic responses [25]. Thermal facial responses are thus probably one component of a thermo-dermal pattern related to the arousal dimension of emotional processing.

Another interesting point is related to the classical negativity bias effect, i.e., a tendency to react faster and stronger to aversive or threatening stimuli than positive or reinforcing ones [51]. In our experiment, the viewing of emotional images evoked a sympathetic response that could be detected through peripheral vasoconstriction and sweat gland activity. It is a nonspecific reaction that goes with an increase in the arousal of the organism that provides the basis for a better analysis or processing of the situation and eventually for a complete emotional reaction to develop. What electrodermal and thermal changes in the nasal area are revealing has to do more with energy mobilisation and preparation to respond than with specific feelings or actual fight/flight responses, in the context of Lang and collaborators’ theory of emotion [4]. That may be the reason why we have not found a negativity bias: the electrodermal and thermal reaction have not been higher to negative than to positive stimuli. As stated above and as shown by researchers from Lang’s and other laboratories, other measures, such as cardiac responses or startle reflex modification, could be more appropriate to index affective valence effects in emotional picture viewing.

## Limitations and conclusions

This study enriched our knowledge in two ways: first, it showed the facial thermal imaging as a salient and reliable marker of emotional arousal; second, it supported the idea that the autonomic signature of emotional arousal consists in two sympathetic cutaneous responses, thermal and electrodermal. The value of these results is reinforced by the rigorous standardization of emotional pictures within the framework of dimensional theory.

In spite of this contribution, it is important to note that emotions elicited by situations other than passive picture watching may elicit different changes. For example, in interpersonal settings, flushing or reddening of the face due to anger or shame in a situation of high arousal cannot be explained by our results. Such limitation should constitute an opportunity to extend the analysis of the thermo-dermal couple and to improve our understanding of facial autonomic output’s emotional neurodynamic. Though in the present study the recording of temperature was circumscribed to the nose, it is important to note that other regions of interest, such as the periorbital area, may yield different thermal imprints because of their direct relationship with fight/flight muscular reactions. More importantly, it should be advisable in further studies to draw a systematic cartography of facial thermal changes and to relate them with other physiological signals known to react to emotions. In a first step, we believe that the dimensional approach of emotion could be an easier way to combine fITI with peripheral or central physiological variables. However, the interesting perspective aiming to establish objective links between fITI and central levels needs an important progress to analyse signals having strongly opposite temporal resolutions.

Finally, the contribution of fITI could be extended and adapted to the social cognition field. Indeed, social interaction situations seem to recruit a different and more complex pattern of thermal variations and their autonomic and muscular associated activity. Social interaction evokes changes in face temperature depending on different variables such as the type of emotion being elicited, interpersonal distance, gaze direction and gender of the people involved in the interaction [8, 9]. Interpersonal situations usually evoke increases in face temperature especially with high arousal, close distance and when the other person is of the opposite gender [8]. The same pattern appears in detection of deception situations [12] with thermal increases. A special case is sympathy crying, elicited by intense emotional feelings of identification with another individual and characterised by a complex autonomic activity pattern that also evokes an increase in face temperature [10]. The potentiality of fITI utilisation in social interactions is coherent with preliminary fITI data we obtained with social vs. non social stimuli in alexithymic and non alexithymic individuals [52]. Overall, thermal facial responses from the tip of the nose can be considered as a reliable indicator of emotional arousal and constitute a credible alternative to the classical electrodermal activity. The slower habituation of the thermal signal could represent a real advantage in studies involving long temporal duration. Finally, the present study provides an original unveiler of autonomic implication in emotional processes and opens a new perspective to measure them in touch-less conditions.
